# Initial experience of transumbilical laparoendoscopic single-site surgery of partial adrenalectomy in patient with aldosterone-producing adenoma

**DOI:** 10.1186/1471-2490-10-19

**Published:** 2010-11-23

**Authors:** Kazuyuki Yuge, Akira Miyajima, Masanori Hasegawa, Yasumasa Miyazaki, Takahiro Maeda, Toshikazu Takeda, Ayano Takeda, Kazutoshi Miyashita, Isao Kurihara, Hirotaka Shibata, Eiji Kikuchi, Mototsugu Oya

**Affiliations:** 1 Department of Urology, Keio University School of Medicine, Tokyo, Japan; 2 Department of Internal Medicine, Keio University School of Medicine, Tokyo, Japan

## Abstract

**Background:**

Laparoscopic single-site surgery has recently emerged in the field of urology and this minimally-invasive surgery has resulted in a further reduction in morbidity compared with traditional laparoscopy. We present our initial experience with laparoendoscopic single-site surgery of partial adrenalectomy (LESS-PA) to treat aldosterone-producing adenomas.

**Case presentation:**

A 60-year-old woman was diagnosed with aldosterone-producing macroadenomas in the left adrenal and aldosterone-producing microadenomas in the right adrenal. A two-step operation was planned. The first step involved transumbilical LESS-PA for the left adrenal tumors. A multichannel port was inserted through the center of the umbilicus and the left adrenal gland was approached using bent instruments according to standard traditional laparoscopic procedures. The tumors were resected using an ultrasonic scalpel, and the resected site was coagulated using a vessel sealing instrument and then sealed with fibrin glue. Operative time was 123 minutes and blood loss was minimal. The patient was discharged from hospital within 72 hours. Her right adrenal microadenomas will be treated in the next several months.

**Conclusions:**

Although our experience is limited, LESS-PA appears to be safe and feasible for treating aldosterone-producing adenomas. More cases and comparisons with the multiport technique are needed before drawing any definite conclusions concerning the technique.

## Background

Laparoscopic surgery has the advantage of a reduced incision size compared with open surgery, resulting in less postoperative pain, a faster recovery, and improved cosmetic outcomes. Current efforts are aimed at further reducing the morbidity of minimally-invasive surgery. Laparoendoscopic single-site surgery (LESS) has emerged as a leading candidate. LESS was first reported in 1998 for cholecystectomy [1] and appendectomy [2]. Initially, LESS did not receive much attention because of technical challenges and a lack of adequate instruments. Recently, these drawbacks have been minimized by advancements in new techniques and instruments, such as multichannel single-access ports, novel bent instruments, and thin flexible laparoscopes. Reports of LESS are increasing in the field of urology, such as for nephrectomy [3-6] and adrenalectomy [7]. Furthermore, a transumbilical approach using LESS was reported to be extremely minimally-invasive because the surgical scar was virtually invisible within the umbilicus, an embryonic natural orifice [8].

Although the current established surgical approach for the adrenal glands is laparoscopy, the safety and feasibility of laparoscopic partial adrenalectomy have been demonstrated in imperative indications, such as bilateral adrenal tumors [9]. There have been a few case reports on elective indications in patients with bilateral aldosterone-producing adenomas treated by laparoscopic partial adrenalectomy on one side and a normal contralateral adrenal gland [10,11]. Herein, we report our initial experience with laparoendoscopic single-site surgery of partial adrenalectomy (LESS-PA), approaching from the umbilicus. To the best of our knowledge, this is the first report of LESS-PA.

## Case presentation

**Figure 1:**
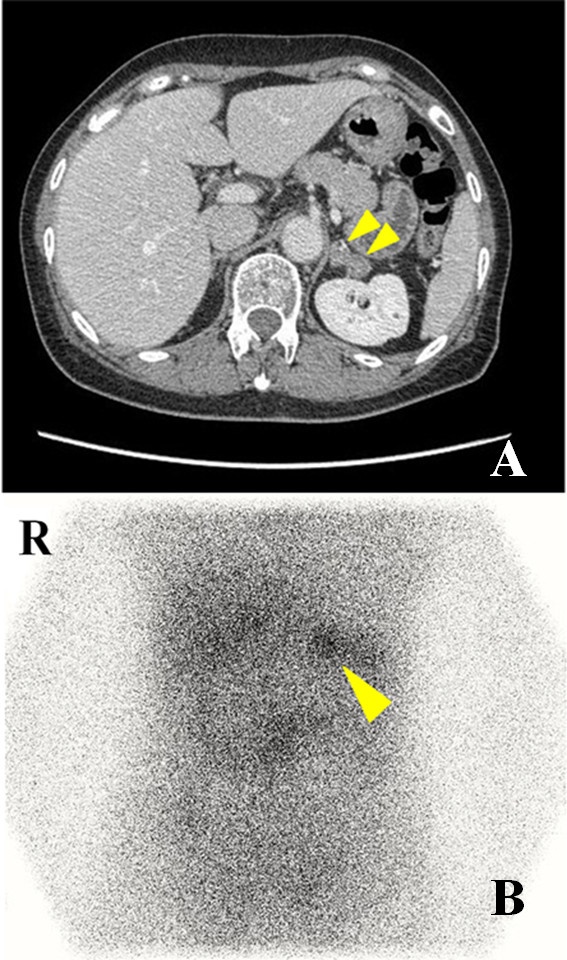
**Abdominal computed tomography and scintigraphy for adrenal glands**. a: Abdominal computed tomography showed two masses in the left adrenal gland: the diameters of the masses were 12 mm and 9 mm. b: Adrenal scintigraphy revealed tracer accumulation in left adrenal regions.

A 60-year-old woman visited our hospital complaining of vertigo. She was hypertensive (169/87 mmHg). Her height was 156 cm, weight 48 kg, and no Cushingoid features were evident. Her complete blood count and serum biochemistry values were within normal limits, including a serum potassium level of 3.7 mEq/L. Endocrinological tests revealed a high urinary aldosterone level (8.1 μg/day), but showed a normal serum aldosterone level (160 pg/mL). Dexamethazone suppression test (1 mg) suppressed endogenous cortisol secretion (2.3 μg/dL). Adrenocorticotropic hormone (ACTH) stimulation testing demonstrated that the peak aldosterone (pg/mL)-to-cortisol (μg/dL) ratio was increased (16.1). The aldosterone (pg/mL)-to-renin (pg/mL) ratio increased following oral captopril administration (45). Furosemide stimulation testing showed decreased renin activity (3.8 pg/mL). Abdominal computed tomography (CT) showed two left adrenal masses with maximum diameters of 12 mm and 9 mm and both masses appeared smooth and well circumscribed (Figure 1a). Iodine-131 aldosterol scintigraphy demonstrated specific uptake in left adrenal regions (Figure 1b). Based on these results, a diagnosis of primary aldosteronism was made. In addition, to localize the laterality of the lesion, an adrenal venous sampling study was subsequently performed. After intravenous injection of ACTH (250 μg), the concentrations of aldosterone on both sides increased (right: 25,600 pg/mL, left: 15,900 pg/mL), and the aldosterone (pg/mL)-to-cortisol (μg/dL) ratio in the right adrenal vein was higher than in the left or inferior vena cava (59.3, 40.0 and 7.9). This suggested that aldosterone was over-produced in the bilateral adrenal glands. She was diagnosed as having aldosterone-producing macroadenomas in the left adrenal and aldosterone-producing microadenomas in the right adrenal. We recommended treating the bilateral aldosterone-producing adenomas with an anti-aldosterone drug, however, the patient expressed a strong desire to have all of the functioning tumors extracted.

**Figure 2:**
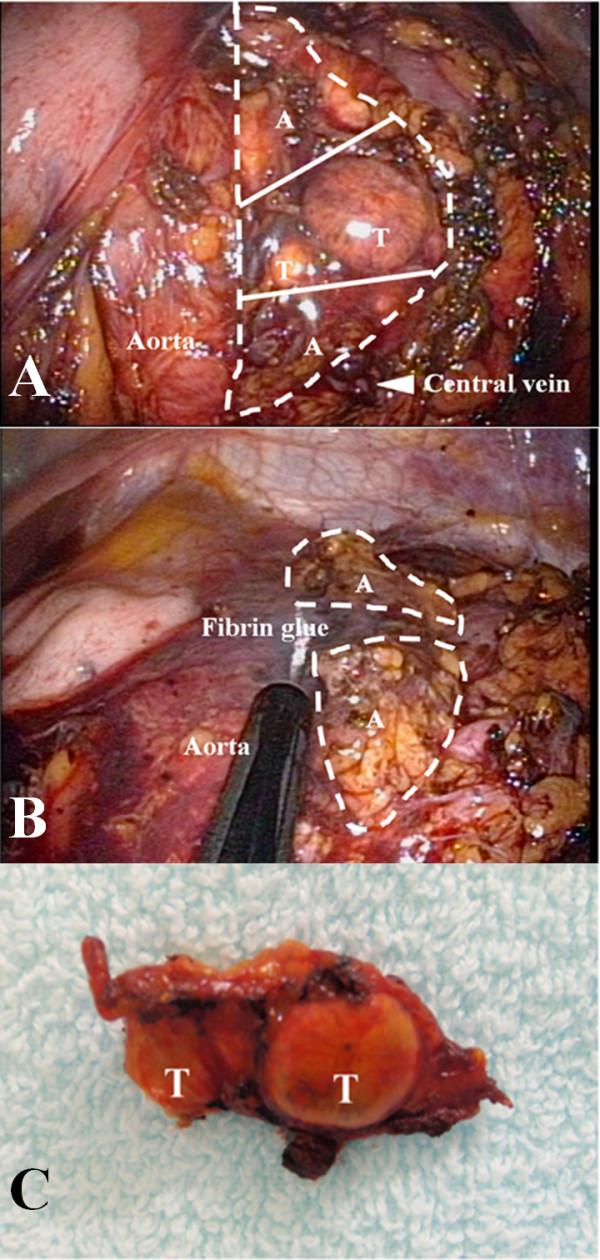
**Perioperative images of adrenal gland and gross appearance of resected tumors**. a: Pre-resection of left adrenal gland. Left partial adrenalectomy. T = tumor; A = normal adrenal tissues. The broken line indicates the shape of the left adrenal gland and the white lines are the resecting lines in the partial adrenalectomy. b: Post-resection of left adrenal gland. The adrenal gland was cut and fibrin glue was applied to prevent hemorrhaging. c: Resected tumors. Aspect of resected tumors of left adrenal gland.

A two-step operation, consisting of left partial adrenalectomy and then right total adrenalectomy, was planned. The left partial adrenalectomy was performed through a single-port by a transumbilical approach. Under general anesthesia, the patient was positioned in the right lateral decubitus 60° flank position. A multichannel port (SILS™port, Covidien, Mansfield, USA) was inserted through the center of the umbilicus. The SILS™port could be placed through a 2 cm incision. A 5 mm flexible laparoscope (Olympus Surgical, Tokyo), Opti4 laparoscopic electrodes (Opti4, Covidien), and bent instruments (Roticulator Endo Mini-Shears, Covidien) were used. The descending colon was dissected from Gerota's fascia and retracted medially. The linorenal and phrenicolienal ligaments were taken down to facilitate medial rotation of the spleen. To expose the hilum of the kidney, the tail of the pancreas was retracted medially. After the left renal vein was found, we identified the central vein and the left adrenal gland, and then removed as much fat as possible. Two xanthous tumors were identified in the center of the left adrenal gland (Figure 2a). The left adrenal gland was separated from the upper pole of the kidney, but not from the surrounding tissue wherever possible in order to preserve the blood supply. While preserving the central vein, the two tumors in the center of the gland were resected using an ultrasonic scalpel (Figure 2b). Both resected sites were coagulated using a vessel sealing device and sealed with fibrin glue. The specimen was removed using an EndoCatch bag (Covidien) (Figure 2c). The operative time was 123 minutes and blood loss was only minimal.

The patient could be discharged from hospital within 72 hours. We planned to treat her right adrenal microadenoma in the months ahead.

We investigated the degree of pain using the visual analog scale (1-10 scale). On the first postoperative day, she experienced pain in the vicinity of her umbilicus (range, 2 to 4). By the second day, however, she was pain-free and did not need an analgesic drug. At discharge, the degree of pain was 1.

From the viewpoint of cosmesis, patient-reported scar satisfaction (1-10 scale) was 10 at one month postoperatively.

## Discussion

Laparoscopic adrenalectomy has been the standard treatment of choice for aldosterone-producing adenoma because of its high success rate, minimal morbidity, and rapid convalescence. Recently, cases of laparoscopic adrenalectomy via single port surgery have been reported [12]. On the other hand, cases of laparoscopic partial adrenalectomy (L-PA) in previous reports required three to four working ports. To the best of our knowledge, this case is the first report of LESS-PA.

The indications for partial adrenalectomy for aldosterone-producing adenoma are still controversial. For example, oral medications, such as an anti-aldosterone drug, are also indicated for the treatment of bilateral primary aldosteronism. In this case, partial adrenalectomy and contralateral total adrenalectomy were discussed at the patient's request.

LESS requires more advanced techniques compared with multi-port laparoscopic surgery because the instruments are introduced adjacent and parallel to each other through a single port and the surgeon has a limited range of motion [13]. The difficulties encountered in LESS mainly arise from the "sword fighting" of the instruments, which perhaps can be reduced by using bent instruments. Wang et al. reported a mean surgical time of 99 minutes (range, 35-196 minutes) in 88 multi-port L-PA cases [14], while Jeschke et al. reported the same mean time of 99 minutes (range, 65-118 minutes) in 13 cases of multi-port L-PA [11]. In the present case, the operative time for LESS-PA was 123 minutes, which was slightly longer than these 2 previous reports of multi-port L-PA. The extra time needed for LESS is due to several reasons. The distance from the port to the tissue in the transumbilical approach is longer than in the conventional laparoscopic approach, and the transumbilical approach becomes more tangential in direction. Moreover, the second- or third-site must be re-grasped because approaching the target tissue in a straightforward manner is difficult in the transumbilical approach. Bent instruments are used to overcome these difficulties, however, there is still room for improvement.

In L-PA, there is a risk of bleeding on the cut surface of the adrenal gland, and hemostasis of the remnant adrenal gland is very important. In the previous reports, the procedure was performed safely by using for example electrocautery and an ultrasonic scalpel to resect the adrenal tumors from normal adrenal parenchyma [10]. Fibrin glue was also used to prevent late hemorrhage from the cut surface. In this case, we cut the adrenal gland using an ultrasonic scalpel, and controlled bleeding from the resected site with a vessel sealing device, and then applied fibrin glue to prevent hemorrhage.

In this case, the low pain scale score and high degree of cosmetic satisfaction with the surgical wound suggest that LESS is a less invasive surgical technique than the conventional method, specifically with respect to enhancement of the cosmetic benefits and reduced wound pain.

## Conclusions

We successfully performed LESS-PA for aldosterone-producing adenomas. Although our experience is still limited, the present case demonstrates the safety and feasibility of transumbilical LESS with hemostatic instruments and agents.

## Consent

Written informed consent was obtained from the patient for publication of this case report and any accompanying images. A copy of the written consent is available for review by the Editor-in-Chief of this journal.

## List of abbreviations used

LESS-PA: laparoendoscopic single-site surgery of partial adrenalectomy, LESS: laparoendoscopic single-site surgery, ACTH: adrenocorticotropic hormone, PA: partial adrenalectomy, CT: computed tomography, L-PA: laparoscopic partial adrenalectomy.

## Competing interests

The authors declare that they have no competing interests.

## Authors' contributions

KY drafted the first manuscript. KY, AM, MH, YM and HS cared for the patient. AM, MH and TM helped to draft the manuscript. All authors reviewed the report and approved the final version of the manuscript.

## Pre-publication history

The pre-publication history for this paper can be accessed here:

http://www.biomedcentral.com/1471-2490/10/19/prepub
